# Increased susceptibility of the left lateral free wall to myocardial delayed enhancement in Duchenne Muscular Dystrophy: progressive systolic dysfunction demonstrable by CMR regional strain analysis

**DOI:** 10.1186/1532-429X-11-S1-P12

**Published:** 2009-01-28

**Authors:** Narayan Kissoon, Kan N Hor, Janaka P Wansapura, Wojciech Mazur, Robert J Fleck, Micheal D Puchalski, D Woodrow Benson, William M Gottliebson

**Affiliations:** 1 University of Minnesota, Minneapolis, MN USA; 2 Cincinnati Childrens Hospital Medical Center, Cincinnati, OH USA; 3 Christ Hospital Medical Center, Cincinnati, OH USA; 4 Primary Childrens Hospital, Salt Lake City, UT USA

**Keywords:** Cardiac Magnetic Resonance, Duchenne Muscular Dystrophy, Duchenne Muscular Dystrophy, Duchenne Muscular Dystrophy Patient, Normal Ejection Fraction

## Background

Cardiac magnetic resonance imaging (CMR) has demonstrated reductions in peak LV myocardial circumferential strain (ε_cc_) despite normal ejection fraction (EF) in Duchenne Muscular Dystrophy (DMD) patients. We hypothesized that the increased initial contractility of the lateral LV free wall makes that region more susceptible to myocardial injury with subsequent fibrosis than the septum in DMD patients.

## Methods

We analyzed regional ε_cc_ from myocardial tagged CMR images on the mid-papillary level LV slice (using HARP™ software) from 14 DMD males with global cardiac dysfunction (LV EF < 55%) and myocardial delayed enhancement (MDE, a marker of myocardial fibrosis), as well as from 13 age-matched control males with normal cardiac function. Regions were assigned based on standard coronary perfusion regions. Regional Δε_cc_ was computed as the difference between normal and DMD subject ε_cc_ per region.

## Results

**Figure 1:**
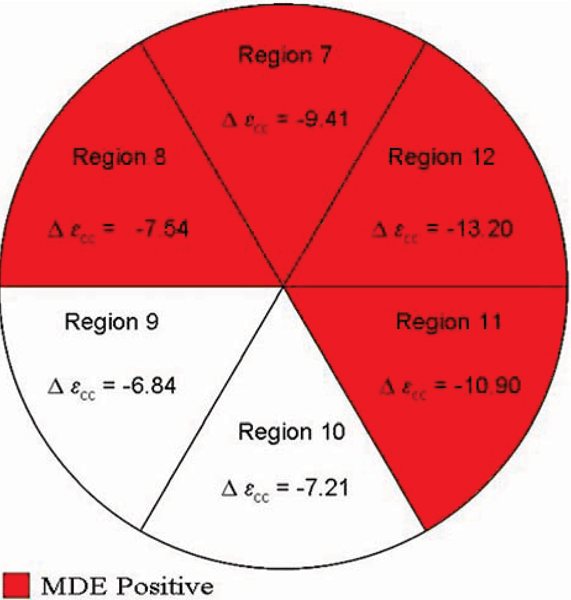
**Regional distribution of MDE and corresponding Δε**_**cc**_.

In controls, the lateral free wall regions had a greater baseline ε_cc_ than the septal regions. In DMD patients, Δε_cc_ was consistently greater in magnitude in the lateral free wall regions when compared to the Δε_cc_ of the septal regions (Figure 1). MDE was consistently detected in these same lateral free wall regions.

## Conclusion

Changes in ε_cc_ show that the regions with greatest contractility in control subjects (the lateral free wall) are the most susceptible to injury in DMD patients, as exemplified both by the greatest reduction in regional ε_cc_ and the development of MDE in those regions.

